# Immunogenicity of small‐cell lung cancer associates with STING pathway activation and is enhanced by ATR and TOP1 inhibition

**DOI:** 10.1002/cam4.5109

**Published:** 2022-08-11

**Authors:** Xuetao Li, Yujun Li, Ziwen Zhao, Nabo Miao, Guorong Liu, Liaoyuan Deng, Shuquan Wei, Jun Hou

**Affiliations:** ^1^ The Laboratory of Computational Medicine and Systems Biology, School of Medicine South China University of Technology Guangzhou Guangdong China; ^2^ Department of Pulmonary and Critical Care Medicine, Guangzhou First People's Hospital, School of Medicine South China University of Technology Guangzhou Guangdong China; ^3^ Department of Pathology, Guangzhou First People's Hospital, School of Medicine South China University of Technology Guangzhou Guangdong China

**Keywords:** cGAS‐STING pathway, DNA damage response, immune infiltration, small‐cell lung cancer, type I IFNs

## Abstract

**Introduction:**

The activation of STING (stimulator of interferon genes) pathway enhances antitumor immunity in small‐cell lung cancer (SCLC), while the DNA damage induced by non‐cGAMP‐based agonists is a potent inducer of STING activity. Here, we investigate the intrinsic expression of STING in cancer cells and evaluate the value of the combination of ATR and TOP1 inhibitors in enhancing antitumor immunity.

**Methods:**

STING expression was assessed at mRNA and protein levels in SCLC and normal lung tissues. Transcriptomic subsets of SCLC were identified based on STING‐related genes. Distinct mutation and immunogenomic profiles of these subsets were determined. The direct antitumor efficacy and the potential of enhancing antitumor immunity of the strategy using the ATR‐TOP1‐inhibitor combination were tested in SCLC cell lines.

**Results:**

The intrinsic expression of STING was significantly reduced in SCLC compared to normal lung tissues (*p* < 0.0001). Three STING‐related SCLC subtypes were identified in which the STING‐high subtype was associated with (1) high immune infiltration, (2) high expression of genes related to MHC and immune checkpoints, and (3) high EMT and ferroptosis score. On the contrary, the STING‐low subtype was enriched with pathways related to DNA damage response (DDR) and cell cycle progression. The association between the DDR pathway activity and the STING‐IFN innate immune response was verified by in vitro experiments in which the inhibition of ATR and TOP1 triggered the expression of genes encoding type I IFN signaling and pro‐inflammatory cytokines/chemokines in a STING‐low SCLC cell line.

**Conclusion:**

Our study verifies that activation of the STING‐IFN response by ATR and TOP1 inhibitors might be a therapeutic strategy to improve the response to immune checkpoint therapy in STING‐low SCLC. Furthermore, the combinations of ATR and TOP1 inhibitors can augment tumor inflammation in STING‐low SCLC.

## Graphical abstract

The combination of ATR and TOP1 inhibitors in STING‐low SCLC triggered activation of the STING‐IFN response and expression of pro‐inflammatory factors, and thus might transform “cold” tumors into “hot” tumors, which are more likely to respond to systemic immunotherapy.

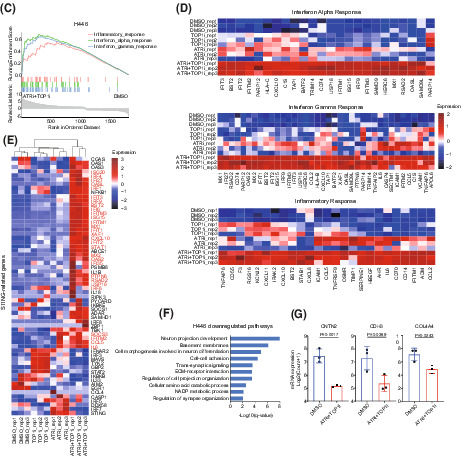

## 1 INTRODUCTION

Small‐cell lung cancer (SCLC), accounting for 15% of all lung cancers, often occurs in heavy smokers and is characterized by the expression of neuroendocrine (NE) markers, rapid cell growth, and early metastatic dissemination.^1^ Although most SCLC patients initially response to first‐line chemotherapy, acquired resistance is inevitable leading to an improved median survival of 10–12 months.^2^ Immune checkpoint inhibitors (ICIs) combined with first‐line chemotherapy (platinum and etoposide) can significantly enhance clinical response of SCLC, but durable response is still scanty despite the fact that most of SCLCs exhibit relative high tumor mutation burden.^3^, ^4^, ^5^, ^6^ The potential mechanisms of immune escape in SCLC may involve the low expression of PD‐L1, downregulation of major histocompatibility complex (MHC) molecules and regulatory chemokines, and less immune infiltration.^7^, ^8^

The specific hallmarks of SCLC include ubiquitously loss of tumor suppressor genes, amplification of oncogenes, and overexpression of transcriptional factors.^9^ Accumulated evidence approved that SCLC is a heterogenous disease consisting of multiple different subtypes.^8^, ^10^, ^11^, ^12^ Gay et al. proposed four SCLC subtypes, SCLC‐A, ‐N, ‐P, and ‐I, defined, respectively, by the high expression of transcription factors *ASCL1*, *NEUROD1*, *POU2F3*, or low expression of all three transcription factor signatures.^11^ Of these, SCLC‐I exhibited high expression of genes related to human leukocyte antigens (HLAs), immune checkpoints, and STING‐induced T‐cell attractant chemokines, such as *CCL5* and *CXCL10*. Moreover, SCLC‐I experienced great benefit from immunotherapy combined with chemotherapy,^11^ while SCLC‐N showed fewer immune infiltration and higher degree of T‐cell dysfunction than SCLC‐A.^8^ These findings casted light on the differences existed in the immune milieu and microenvironment between SCLC subtypes. However, conflicting concepts were also reported which described SCLC‐I subtype as an immunosuppressive tumor.^11^ Therefore, it is necessary to incorporate additional immune components when classifying SCLC.

The cyclic GMP‐AMP synthase (cGAS)‐STING signaling not only plays a pivotal role in the host defense against microbial infection but also modulates tumorigenesis.^13^ The binding of cytosolic double‐stranded DNA (dsDNA) and its sensor cGAS activates STING and then recruits TANK‐binding kinase 1 (TBK1), a critical downstream regulator of innate immune signaling, to stimulate interferon regulatory factor 3 (IRF3), thereby induces production of type I IFNs, arouses T‐cell responses, and thus facilitates innate immune signaling.^13^, ^14^ Recently few studies revealed that upregulated cGAS‐STING signaling facilitates the recruitment of tumor‐infiltrating CD8^+^ T cells by promoting the production of IFNβ and pro‐inflammatory chemokines such as *CCL5* and *CXCL10*.^15^, ^16^ *STING* (also known as *TMEM173*) has emerged as a potential target for cancer therapy. STING agonist enhances response to anti‐PD‐1/PD‐L1 agents by promoting CD8^+^ T‐cell infiltration and increasing expression of MHC molecules on tumor cell.^17^ The presence of dsDNA also triggers formation of the Absent in Melanoma 2 (AIM2) inflammasome and then promotes the activation of caspase‐1 (CASP1), which in turn induces the production of pro‐inflammatory cytokines (IL‐1β and IL‐18).^13^

DNA damage response (DDR) pathway is frequently disrupted during cancer development and progression. Overexpression of DDR‐related proteins in SCLC, such as poly (ADP‐ribose) polymerase 1 (PARP1) and checkpoint kinase 1 (CHK1) has been reported.^18^ More recently, the potential of DDR inhibitors as monotherapy or in combination with other therapies has been explored in preclinical SCLC models and SCLC patients. For example, promising clinical outcome was achieved by PARP1 inhibitor olaparib in combination with chemotherapeutic agent temozolomide in relapsed SCLC, presenting a great improvement compared to the modest response induced by DDR inhibitors as a single agent.^19^ Also, Ataxia Telangiectasia and Rad3‐related (ATR) protein is an essential kinase in the DDR transduction signaling pathway. It has been shown that the combination of ATR inhibitor and Topotecan led to substantial clinical benefit and durable responses in platinum‐resistant SCLC patients.^20^, ^21^ An important study showed that treatment with PARP1 or CHK1 inhibitor enhanced response to PD‐L1 blockade through boosting STING‐mediated T‐cell effects in SCLC.^22^ The exposure to DNA‐damaging stimulants such as clinical chemotherapy and radiotherapy, as well as DDR inhibitors can increase cytoplasmic DNA and lead to activation of cGAS‐STING pathway and production of type I IFNs.^23^ However, the antitumor effect exerted by the combination of ATR inhibitor with topoisomerase (TOP1) inhibitor and the subsequent mechanisms underlying enhanced tumor immunogenicity in SCLC remain poorly understood. Also, little is known about the intrinsic expression level of STING in SCLC tumor.

In this study, we implemented systematic characterization of STING pathway in SCLC tumors, in which we determined that STING expression was downregulated in SCLC tumors compared to normal lung tissues and identified a STING‐high SCLC subtype associated with increased expression of immune‐related genes and decreased NE score. We also investigated the potential effects of combining ATR (M4344/VX‐803) and TOP1 (topotecan) inhibitors in triggering immune response signaling and oncogenic signaling pathways in SCLC cells, which could provide a new therapeutic strategy for SCLC.

## 2 MATERIALS AND METHODS

### 2.1 Data acquisition

All microarray data were downloaded from Gene Expression Omnibus (GEO, RRID:SCR_005012). The GSE30219^24^ is a set of microarray data for lung cancer samples with different histological subtypes. The GSE149507^25^ is a set of microarray data for human SCLC tumors and matched normal lung tissue. The GSE43346^26^ is a set of microarray data for human SCLC tumors. The GSE168266^27^ is a set of microarray data that examines the different expression of MHC Class I in human SCLC tumors. In addition, RNA‐seq FASTQ files of 59 human SCLC tumors, whole‐genome sequencing (WGS) BAM files of 54 primary SCLC tumors, and paired normal samples were obtained from the University of Cologne and downloaded from the European Genome‐phenome Archive (EGA, RRID:SCR_004944) under accession code EGAS00001000925.^9^ Gene expression data of 50 SCLC human cell lines were taken from the Cancer Cell Line Encyclopedia (CCLE, https://portals.broadinstitute.org/ccle).

### 2.2 Cell lines and compounds

Human SCLC cell lines NCI‐H446, NCI‐H146, SHP‐77, and human NSCLC cell line A549 were kindly provided by Stem Cell Bank, Chinese Academy of Sciences. M4344 (VX‐803, ATR inhibitor, Selleck, Cat.# S9639), and Topotecan (TOP1 inhibitor, Selleck, Cat.# S9321) were purchased from Selleck.cn.

### 2.3 Gene expression data processing

#### 2.3.1 Microarray data (GEO cohort)

Raw CEL files were processed by MAS5.0 normalization. Human SCLC samples from GSE30219 (*n* = 21), GSE149507 (*n* = 18), and GSE43346 (*n* = 23) were integrated as GEO cohort. Batch effects were removed using “ComBat” function in the “sva” R package. Probeset IDs were converted to gene symbols based on the corresponding gene annotations (GPL570). For each sample, the measurements without a gene annotation were excluded.

#### 2.3.2 Human RNA‐Seq data (George_2015 cohort)

FASTQ files were trimmed using Trimmomatic^28^ to improve read alignments. Paired‐end reads were aligned to the Human genome (ENSEMBL CRCh38) using STAR (v2.7.3a).^29^ Gene expression was subsequently quantified using RSEM^30^ (v.1.3.3). We removed genes whose FPKM was 0 in more than 75% of the samples and the expression value of the remaining genes was transformed by log2[FPKM+1] for further analysis.

### 2.4 SCLC classification

Genes were selected from KEGG: hsa04623, “Cytosolic DNA‐sensing pathway”, KEGG NETWORK: N00395, “cGAS‐STING signaling pathway”, and Reactome: R‐HSA‐909733, “Interferon alpha/beta signaling”. Genes were not included in gene set when the expression value of genes was 0 in more than 75% of the samples. A total of 62 key STING signaling‐related genes were selected to define SCLC subclasses. The gene list is summarized in Table S1–S4. Hierarchical clustering analysis was performed to cluster SCLC samples in each cohort based on STING signaling‐related genes signature.

### 2.5 Calculation of NE score, EMT score, and ferroptosis score

The 50 genes of the NE signature have been described by Zhang et al.^31^ A quantitative NE score can be generated from this signature using the formula: NE score = (correl NE − correl non‐NE)/2, where correl NE (or non‐NE) is the Pearson correlation between expression of the 50 genes in the test sample and expression of these genes in the NE (or non‐NE) cell line. This score has a range of −1 to +1, where a positive score predicts for NE, while a negative score predicts for non‐NE cell types. EMT scores were calculated for all SCLC subjects based on 200 EMT‐related genes from MSigDB (https://www.gsea‐msigdb.org/gsea/msigdb) using single‐sample gene‐set enrichment analysis (ssGSEA) method. Ferroptosis scores were calculated based on 45 ferroptosis‐related genes from a previously published study^32^ using ssGSEA. The gene lists are summarized in Table S2.

### 2.6 Tumor‐infiltrating immune cell profile analysis

We employed current acknowledged algorithms such as ssGSEA, xCell, and quanTIseq to investigate the tumor‐infiltrating immune cell landscape of SCLC samples. Firstly, we applied ssGSEA to assess the level of 29 immune infiltrates according to the expression levels of given immune cell‐specific marker genes. Marker genes for each immune cell type were obtained from a recent publication.^33^ The ssGSEA scores were calculated using the GSVA method.^34^ xCell, a novel gene signature‐based method, was used to infer 64 immune and stromal cell types.^35^ The quanTIseq^36^ is a deconvolution algorithm to estimate the proportions of 10 different immune cell types from bulk transcriptomic data.

### 2.7 Whole‐genome sequencing data analysis

Sorting and indexing of BAM files used SAMtools (v1.9) and PCR duplicate reads were removed using Picard (v2.20.1). Single nucleotide variants (SNVs) were identified for tumor and matched normal samples with VarScan2 (v2.3.9) using the pileup files created by SAMtools mpileup. SNVs and small insertions/deletions (indels) calls were annotated using the UCSC hg19 database with ANNOVAR. The refGene, 1000 Genomes (2015_08), COSMIC (v70), and avSNP 150 were also used for the variant annotation. The somatic variant calls were excluded with the following steps: (1) removing variants with VAFs lower than 0.1, (2) filtering all intronic and UTR variants, (3) removing all variants identified by the 1000 genomes project, (4) germline variants detected in the matched normal tissues were excluded from each tumor variant results.

### 2.8 RNA sequencing and expression analysis from cell lines

RNA was isolated directly from cell line samples with the QIAGEN (Hilden) RNeasy isolation kit using on‐column DNAse digestion. RNA quality was analyzed using Agilent 5400 bioanalyzer, and only samples with RIN >8 were used. RNA libraries were prepared from 100 ng of total RNA using the NEBNext® Ultra™ RNA Library Prep Kit per manufacturer's instructions. RNA sequencing was performed on Illumina NovaSeq 6000 machines (Illumina) using the standard Illumina RNA‐seq protocol with a read length of 2 × 150 bases.

Raw RNA‐seq reads were aligned to the reference genome (ENSEMBL CRCh38) using STAR^29^ (v.2.7.3a). Gene expression was subsequently quantified using RSEM^30^ (v.1.3.3). Differential gene expression analysis for RNA‐seq data was performed using DESeq2.^37^ Genes with |log2 fold change| > 1 and Benjamini–Hochberg adjusted *p*‐values <0.05 were considered significantly differentially expressed. GSEA was performed on the lists of differentially expressed genes using the Hallmark gene sets from MSigDB v 7.4.^38^ We considered pathways with Benjamini–Hochberg adjusted p‐values <0.05 to be significant. Visualization of the results and downstream analyses were performed using R software.

### 2.9 Immunohistochemistry staining and image analysis

Samples archived between 2019 and 2021 were retrospectively collected from Guangzhou First People's Hospital, including SCLC (*n* = 30), lung adenocarcinoma (*n* = 10), lung squamous cell carcinoma (*n* = 10) tumor samples, and normal lung tissues (*n* = 10). The samples were surgical resections or biopsies preserved as formalin‐fixed paraffin‐embedded (FFPE) blocks and prepared following standard procedure. FFPE tissues were cut into 4 μm thick serial sections for IHC staining of STING (1:500 dilution, #13647, Cell Signaling Technology) and CASP1 (1:100 dilution, #sc‐56,036, Santa Cruz Biotechnology). Slides were deparaffinized and rehydrated through a series of washes of graded ethanol to deionized water. Antigen retrieval was performed by heat treatment of the deparaffinized sections in a pressure cooker in EDTA (PH8.0) for 3 min. Slides were then stained with primary antibodies for 40 min at 37°C. After incubation with secondary antibody at 37°C for 40 min, the sections were washed again and visualized by 3, 3′‐diaminobenzidine (DAB) chromogen detection kit. Finally, slides were counterstained with hematoxylin, dehydrated in graded alcohol and xylene, and mounted with EcoMount mounting medium. All stained slides were evaluated by one experienced pathologist. The staining intensity was sorted by 0 (negative), 1 (weak), 2 (moderate), and 3 (strong). Depending on the staining extent, the area was categorized as 0 (<5%), 1 (5%–25%), 2 (26%–50%), 3 (51%–75%), and 4 (>75%). The IHC score was computed by multiplying staining intensity with staining extent score.

Written informed consent to medical research on identifiable data was obtained from all included patients. This study was reviewed and approved by the Ethical Review Committee for Biomedical Research, Guangzhou First People's Hospital. The study design and all procedures were conducted in accordance with the Declaration of Helsinki.

### 2.10 Cell viability assay and cell line samples collection

All cell lines were cultured in Roswell Park Memorial Institute (RPMI) 1640 media supplemented with 10% fetal bovine serum (FBS) and 1% penicillin/streptomycin. All experiments were performed before reaching 15 passages. Cells were seeded into the internal wells of 96‐well plates at a density of 5 × 10^3^/well and incubated in a 37°C/5%CO_2_ incubator. Outer wells contained PBS only without cells. We first profiled drug concentrations for combination by treatment with the M4344 and Topotecan alone. M4344 was added to wells to achieve six final concentrations of (1, 0.5, 0.25, 0.125, 0.0625, 0.03125, 0.015625 μM). Topotecan was added to wells to achieve six final concentrations of (1, 0.5, 0.25, 0.125, 0.0625, 0.03125, 0.015625 μM). And then, a low toxic dose (20 nmol/L) of Topotecan was used for combination treatment. All cells were cultured for 48 h in a media containing the drugs or with dimethyl sulfoxide (DMSO) as control. Cell viability was measured by Cell Counting Kit‐8 (CCK‐8) (Biosharp, # BS250B) assay to evaluate cytotoxicity of drugs.

Cell lines were treated with M4344, Topotecan, and the two‐agent combination inhibition, respectively, dissolved in culture medium that was prepared. After 48 h, cells were washed and replenished with fresh culture medium (without inhibitors) and rested for an additional 48 h before collecting.

### 2.11 Statistical analysis

All data were presented as mean ± SD. Means for all data were compared by one‐way ANOVA or unpaired *t*‐test or Mann–Whitney *U* test. The statistical significance was defined as *p*‐value <0.05. Statistical analyses were performed using R (version 3.6.3, https://www.r‐project.org/), SPSS (version 26), and GraphPad Prism (version 8).

## 3 RESULTS

### 3.1 Expression of STING signaling‐related genes is decreased in SCLC

**FIGURE 1:**
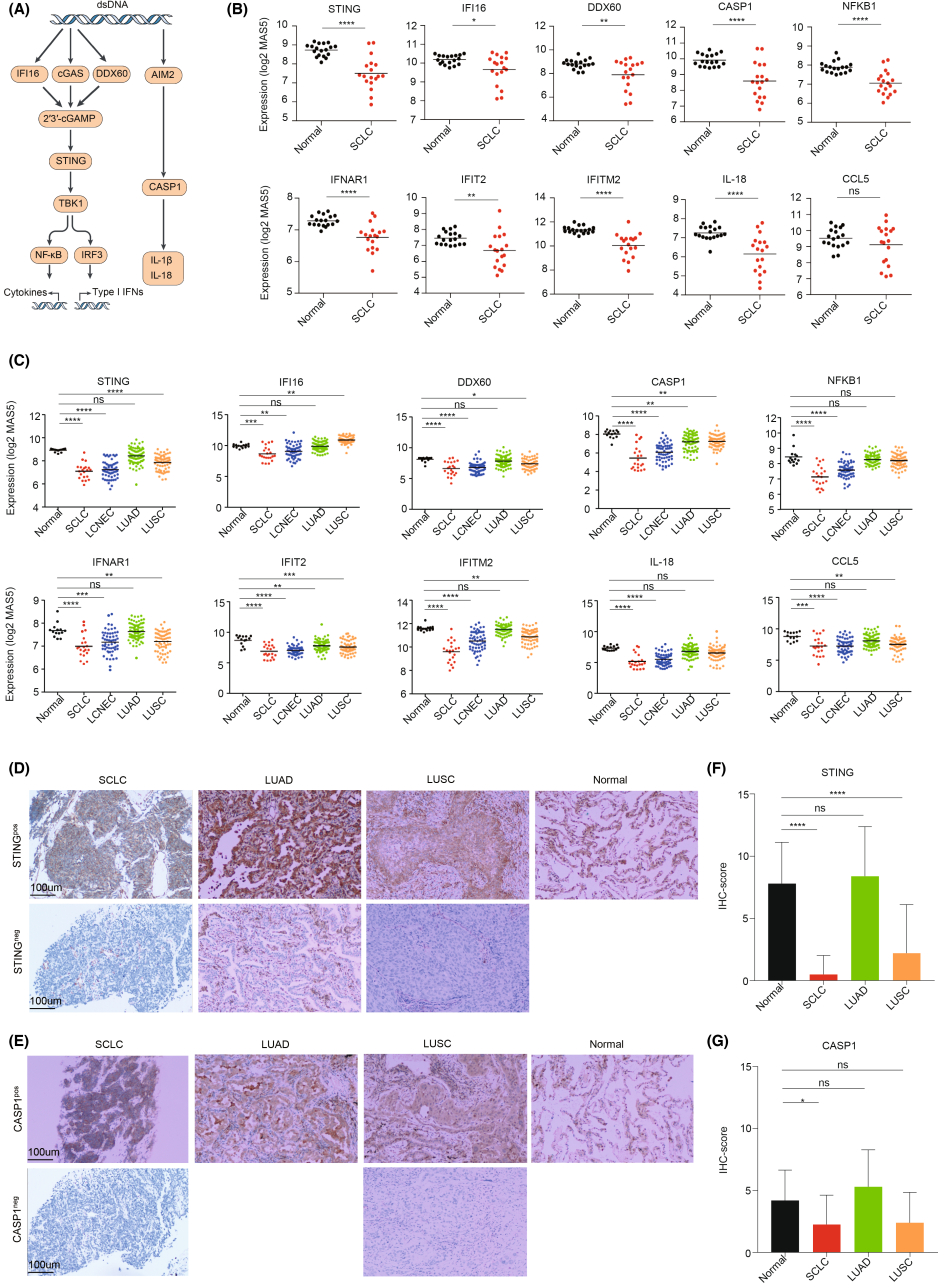
Expression of STING signaling‐related genes is reduced in SCLC. (A) Schematic of dsDNA sensing pathways that activate STING pathway and induce type I IFNs. (B) Relative mRNA expression of STING signaling‐related genes in SCLC and paired normal lung tissues. (C) Relative mRNA expression of STING signaling‐related genes among different histological subtypes in the lung. (D, E) Representative IHC staining images of *STING* (D) and *CASP1* (E) in SCLC, LUAD, LUSC, and normal lung tissues. (F, G) IHC score of *STING* (F) and *CASP1* (G) expression in SCLC, LUAD, LUSC, and normal lung tissues. LCNEC, large cell neuroendocrine cancer; LUAD, lung adenocarcinoma; LUSC, lung squamous cell carcinoma; ns, not significant. *p* values were calculated by unpaired *t*‐test (B) and one‐way ANOVA (C, F, and G). *p* values of statistical significance are represented as *p < 0.05, **p < 0.01, ***p < 0.001, and ****p < 0.0001.

The activation of STING pathway is typically regulated by dsDNA sensing and the downstream effect includes the induction of type I IFN responses and innate immune signaling. We examined the expression of key genes along these signaling axes (Figure 1A). Our analysis revealed a universal downregulation of STING signaling‐related gene expression in SCLC samples compared with normal lung tissues, including genes involved in dsDNA sensing (*IFI16* and *DDX60*), STING pathway (*STING*), inflammasome (*CASP1*), pro‐inflammatory factors (*NFKB1*, *CCL5*, *IL‐18*), and type I IFN response (*IFNAR1*, *IFIT1/2/3*, *IFITM2*) (Figure 1B,C, Figure S1A,B). Interestingly, most of these genes were also significantly downregulated in lung large cell neuroendocrine cancer (LCNEC) and lung squamous cell carcinoma (LUSC), but not in lung adenocarcinoma (LUAD) (Figure 1C, Figure S1B). Remarkably, the levels of *cGAS*, which directly binds cytoplasmic dsDNA and is upstream of STING, were unaltered in SCLC cells compared with normal lung (Figure S1A,B), suggesting cGAS‐independent STING pathways may play important roles in SCLC. Similarly, SCLC cell lines had low expression levels of *STING* and *CASP1* across most cancer cell lines from CCLE dataset (Figure S1C). Together, these data confirmed that the expression of most STING‐related genes was decreased in cancer lesions, especially SCLC.

To further validate these findings in mRNA expression level, intrinsic *STING* and *CASP1* protein levels were evaluated by IHC across SCLC, LUAD, LUSC tumor samples and normal lung tissues. *STING* and *CASP1* were mainly detected in the cell cytoplasm and can be observed in cancer cells and surrounding stroma (Figure 1D,E). The majority of SCLCs (*n* = 24, 80%) displayed no *STING* expression (STING^neg^); whereas 20% (*n* = 6) of SCLC cases exhibited positive *STING* expression (STING^pos^) (Table 1). We detected that *STING* protein expression was higher in tumor with LUAD (median score = 7.8) than in SCLC (median score = 0.5) and LUSC (median score = 2.2) (Figure 1F). Consistent with *STING* mRNA levels, STING protein levels were also significantly reduced in SCLC and LUSC tissues, in comparison with the normal lung tissues (*p* < 0.0001) (Figure 1A,B,F). However, no differences in expression of *STING* were observed between LUAD and normal lung tissues. *CASP1* expression was significantly lower in SCLC than in normal lung tissue (*p* = 0.0315) (Figure 1G). We also found that almost all LUAD tissue specimens were positive for *CASP1* staining.

**TABLE 1:** Baseline clinical characteristics of SCLC patients

Variable	Total (*n* = 30) (100%)	STING^pos^ (*n* = 6) (20%)	STING^neg^ (*n* = 24) (80%)	*p* value
Age				
Median (y)	64.5 (50–90)	71 (63–90)	62 (50–82)	
<65	15 (50)	1 (16.7)	14 (58.3)	0.171
≥65	15 (50)	5 (83.3)	10 (41.7)	
Gender				
Male	29 (96.7)	6 (100)	23 (95.8)	
Female	1 (3.3)	0 (0)	1 (4.2)	
Stage				0.424
Limited stage	6 (20)	0 (0)	6 (25)	
Extensive stage	24 (80)	6 (100)	18 (75)	
Tumor size				
≤5 cm	10 (33.3)	1 (16.7)	9 (37.5)	
>5 cm	13 (43.3)	4 (66.7)	9 (37.5)	
Unknown	7 (23.3)	1 (16.7)	6 (25)	
TNM stage				
I	0 (0)	0 (0)	0 (0)	
II	1 (3.3)	0 (0)	1 (4.2)	
III	8 (26.7)	1 (16.7)	7 (29.2)	
IV	21 (70)	5 (83.3)	16 (66.7)	
T stage				
T1	0 (0)	0 (0)	0 (0)	
T2	2 (6.7)	0 (0)	2 (8.3)	
T3	2 (6.7)	0 (0)	2 (8.3)	
T4	26 (86.7)	6 (100)	20 (83.3)	
N stage				0.849
N1	1 (3.3)	0 (0)	1 (4.2)	
N2	18 (60)	4 (66.7)	14 (58.3)	
N3	11 (36.7)	2 (33.3)	9 (37.5)	
M stage				0.765
M0	9 (30)	1 (16.7)	8 (33.3)	
M1	21 (70)	5 (83.3)	16 (66.7)	
TTF‐1				0.156
—	4 (13.3)	1 (16.7)	3 (12.5)	
1+	14 (46.7)	5 (83.3)	9 (37.5)	
2+	2 (6.7)	0 (0)	2 (8.3)	
3+	10 (33.3)	0 (0)	10 (41.7)	
Syn				0.547
**—**	0 (0)	0 (0)	0 (0)	
1+	20 (66.7)	5 (83.3)	15 (62.5)	
2+	3 (10)	0 (0)	3 (12.5)	
3+	7 (23.3)	1 (16.7)	6 (25)	
CgA				0.227
—	1 (3.3)	1 (16.7)	0 (0)	
1+	22 (73.3)	4 (66.7)	18 (75)	
2+	1 (3.3)	0 (0)	1 (4.2)	
3+	6 (20)	1 (16.7)	5 (20.8)	
CD56				
—	1 (3.3)	1 (16.7)	0 (0)	
1+	9 (30)	3 (50)	6 (25)	
2+	1 (3.3)	0 (0)	1 (4.2)	
3+	9 (30)	0 (0)	9 (37.5)	
Unknown	10 (33.3)	2 (33.3)	8 (33.3)	
CK				
—	0 (0)	0 (0)	0 (0)	
1+	16 (53.3)	5 (83.3)	11 (45.8)	
2+	2 (6.7)	0 (0)	2 (8.3)	
3+	3 (10)	1 (16.7)	2 (8.3)	
Unknown	9 (30)	0 (0)	9 (37.5)	

*Note*: Values were shown as *n* (%) unless otherwise noted. *p*‐value: Fisher exact test. Characteristics with unequal distribution and too many missing data were not assessed in statistical analysis.

Abbreviation: CK, cytokeratin.

Of note, due to small sample size and unequal distribution of patient baseline characteristics, such as gender (Table 1), the associations between the expression of *STING* and clinical parameters of SCLC patients need to be further assessed in big patient cohorts.

### 3.2 STING pathway expression stratifies distinct SCLC molecular subtypes

**FIGURE 2:**
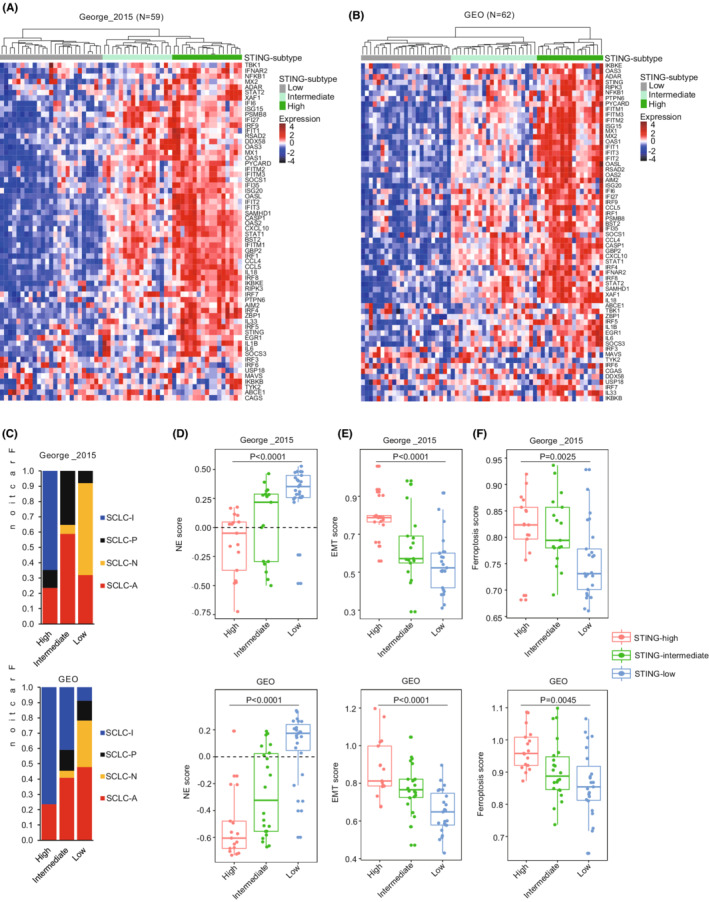
Hierarchical clustering analysis identifies three STING‐related SCLC subtypes. (A, B) Hierarchical clustering analysis was performed to cluster SCLC samples based on expression of STING signaling‐related genes in George_2015 (A) and GEO (B) cohorts. (C) Correlation between STING subtypes described here and previous four transcription factor‐defined SCLC subtypes. (D–F) Subtype‐specific NE score (D), EMT score (E), and ferroptosis score (F) were calculated for each SCLC tumor with comparison between mean scores for each subtype. *p* values were calculated by one‐way ANOVA (D–F).

To get an overall view of STING‐pathway activity across SCLC, hierarchical clustering analysis based on 62 STING‐related genes was performed on previously published SCLC RNA‐seq dataset (George_2015 cohort). We identified three distinct subtypes—STING‐high, STING‐intermediate, and STING‐low (Figure 2A). Similarly, these subtypes were recapitulated in an independent cohort composed of 62 RNA microarray data of human SCLC tumor samples (GEO cohort) (Figure 2B). We next assessed the relationship between these three STING subtypes and four SCLC subtypes previously established based on the expression of transcription factors. Interestingly, the majority of SCLC‐N samples fall within the STING‐low subtype, while the SCLC‐A samples distributed across all STING subtypes (Figure 2C, Figure S2). As expected, we observed that SCLC‐I samples were mainly assigned to STING‐high and ‐intermediate subtypes (Figure 2C). Taken together, we defined a new SCLC molecular classification, which was not exactly the same as SCLC subtypes defined by three transcription factors.

### 3.3 STING‐high SCLC exhibits low level of NE, high potential of EMT, and ferroptosis

SCLC is the most common form of neuroendocrine lung cancer and classic SCLC expresses SCLC‐specific NE markers. Using a previously validated NE score wherein more positive values indicate higher levels of NE differentiation,^31^ we found that STING‐low SCLC had a higher NE score compared to STING‐high and ‐intermediate SCLC (Figure 2D). Epithelial‐mesenchymal transition (EMT) has been previously reported to be related to non‐NE SCLC.^11^ Using ssGSEA, we developed a computation of EMT scores based on EMT‐related genes and assessed the degree of EMT for individual SCLC samples. STING‐high SCLCs had higher EMT score than STING‐low subjects (Figure 2E). Recently, ferroptosis has been reported as a vulnerability specifically found in non‐NE‐subtype SCLC.^39^ A ferroptosis score was constructed using ssGSEA method based on 45 ferroptosis‐related genes^32^ and employed in the current study to estimate the ferroptosis in individual SCLCs. Strikingly, the lowest ferroptosis scores were found in STING‐low SCLCs, suggesting that ferroptosis escape may exist in this subtype SCLC (Figure 2F).

### 3.4 STING‐high SCLC exhibits high level of immunogenicity and increased immune infiltration

**FIGURE 3:**
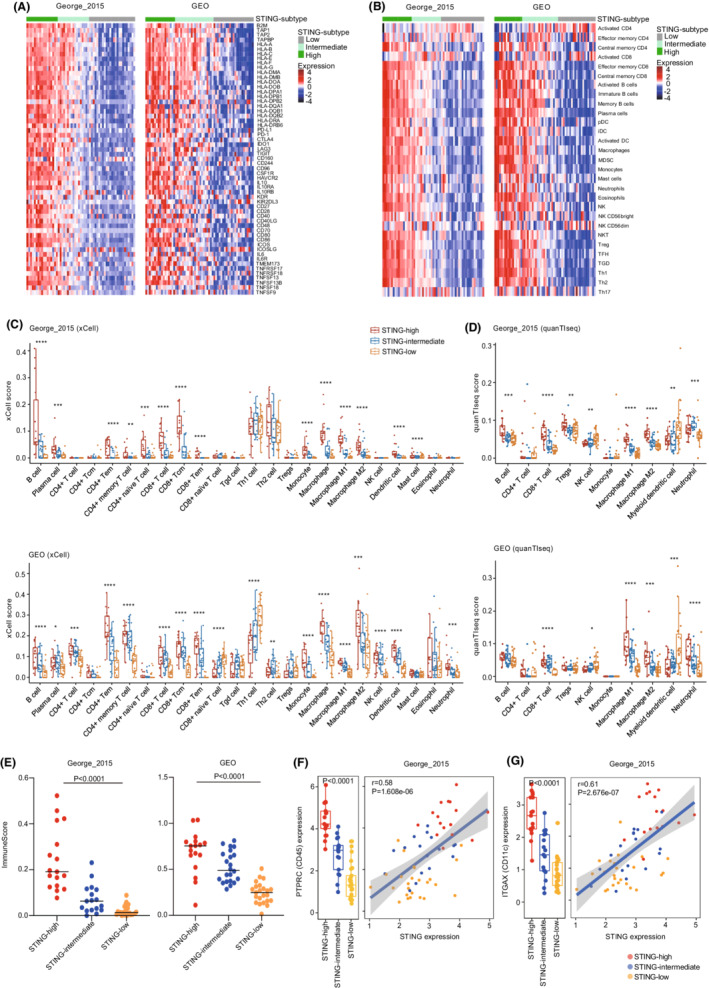
STING‐high defines an inflamed subtype of SCLC. (A) Heatmaps comparing expression of HLA and antigen‐presenting genes, immune checkpoints, and immunomodulatory factors across three SCLC subtypes. (B) Heatmaps comparing immune infiltrations according to ssGSEA score across three SCLC subtypes. (C, D) The distribution of immune cell populations across three SCLC subtypes. The xCell (C) and quanTIseq (D) methods were used. (E) Comparison of ImmuneScore from xCell. (F, G) Box whisker plots show the levels of *PTPRC* (F) and *ITGAX* (G) in different STING‐related SCLC subtypes from George_2015 cohort. Scatterplots show the correlation between expression of *STING* and *PTPRC* or *ITGAX*. Color for scatterplot symbols reflects different subtypes. *p* values were calculated by Mann–Whitney *U* test (C and D), One‐way ANOVA (E–G for box whisker plots), and Pearson correlation analysis (F and G for scatterplots). *p* values of statistical significance are represented as *p < 0.05, **p < 0.01, ***p < 0.001, and ****p < 0.0001.

SCLC has been labeled as immune‐cold cancer, displaying intrinsically low expressions of MHC‐I and few immune infiltration.^8^, ^27^ However, recent findings also suggested that high degrees of immunogenicity existed in a subset of SCLC, mainly non‐NE, which were characterized by abundant infiltration of T cells, NK cells, and macrophages.^11^, ^40^ Here, we aimed to assess the relationship between STING subtypes and tumor immunogenicity in SCLC and observed markedly higher expression of immune‐related genes, including HLAs and immune checkpoints, in STING‐high compared with STING‐low SCLC tumors (Figure 3A). This immunogenic pattern was further verified by the observed positive correlation between STING‐signature genes and MHC‐I protein expression. We found that most of the STING‐related genes had significantly higher expression in MHC‐I high SCLCs compared to MHC‐I low SCLCs (Figure S3A).

To further assess immune context in SCLC STING subtypes, we used different computational tools including ssGSEA, xCell, and quanTIseq to infer and deconvolute from bulk transcriptome data the immune compositions in SCLC. Compared with STING‐low SCLCs, STING‐high tumors exhibited significantly higher immune infiltrates and ImmuneScore, especially B cells, T cells, macrophages, and dendritic cells (Figure 3B–E). Furthermore, *STING* expression positively correlated with *PTPRC* (which encodes pan‐leukocyte marker CD45), and the highest *PTPRC* gene expression was found in STING‐high SCLC tumors (Figure 3F and Figure S3B). Also, the *ITGAX* (which encodes for dendritic cell marker CD11c), *CD8A* (a marker of CD8^+^ T cell), and *MS4A1* (which encodes for B‐cell marker CD20) genes presented markedly high expression in STING‐high SCLC tumors, and they positively correlated with *STING* expression (Figure 3G, Figure S3C,D). Collectively, these findings suggested that STING‐high SCLC tumors are highly immunogenic and characterized by increased immune infiltrates.

### 3.5 STING‐low SCLCs display marked enrichment of pathways associated with cell cycle progression and DNA damage response

**FIGURE 4:**
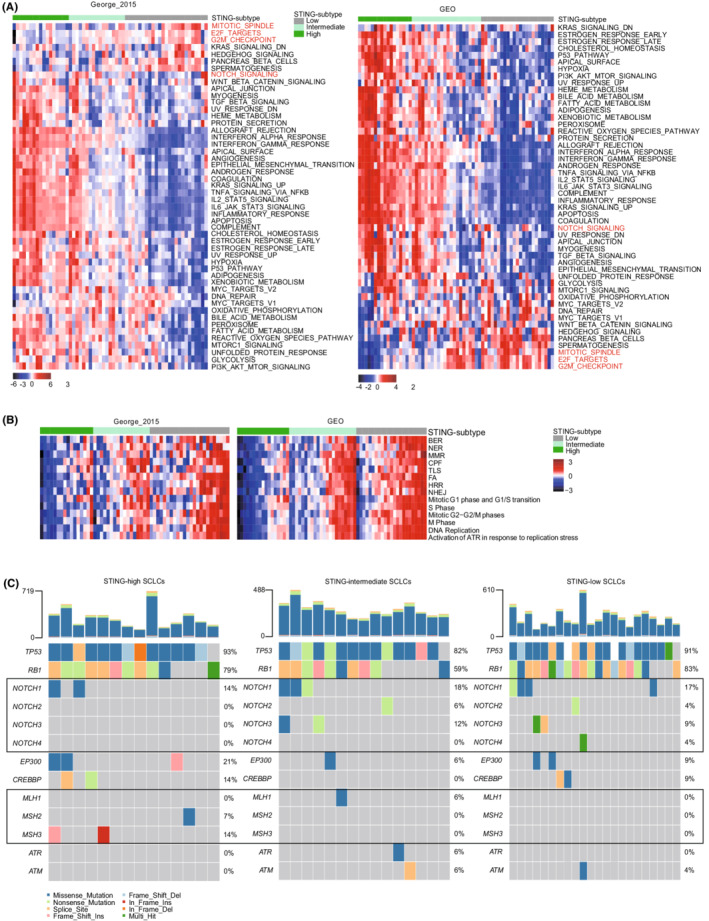
Activated pathways and mutation profiles in STING subtypes. (A) Heatmaps of GSEA normalized enrichment score (NES) for hallmark gene sets among three SCLC subtypes. (B) Heatmaps of pathways involved in DNA damage response and cell cycle regulation. (C) Mutation profiles in SCLC‐specific genes and in DNA damage response‐associated genes in each STING‐related SCLC subtype. BER, base excision repair; CPF, checkpoint factor; FA, Fanconi anemia; HRR, homologous recombination repair; MMR, mismatch repair; NER, nucleotide excision repair; NHEJ, non‐homologous end joining; TLS, DNA translesion synthesis.

To determine the biological processes distinctly activated in individual SCLC STING subtypes, enriched gene pathways were identified using GSEA based on 50‐hallmark gene sets in the GSEA database. While immune‐related pathways presented low enrichment scores in STING‐low SCLCs, the pathways linked to cell cycle progression were obviously enriched in this subtype, including E2F_targets, G2M_checkpoint, and mitotic_spindle (Figure 4A), suggesting that STING‐low SCLCs displayed a low immunogenicity, but a high proliferation ability. We next set to investigate the relationship between the activation of STING pathway and cellular processes involved in DDR, DNA replication, and cell cycle progression. The observation included that STING‐low SCLC tumors exhibited activation of DDR, DNA replication, and cell cycle progression‐related pathways (Figure 4B), and their activation has been linked to high replication stress in previous studies.^20^, ^41^ Inactivation of DDR pathways was a feature of STING‐high SCLCs, which coincides with previous studies showing impaired DDR program was associated with activated cGAS‐STING pathway.^42^

Further, we tested whether these three distinct STING subtypes were associated with specific genomic alterations. The prevalence of *TP53* and *RB1* mutations showed no difference across STING subtypes, while *NOTCH* mutation was found more frequent in the STING‐low (7/23, 30.4%) and STING‐intermediate SCLCs (5/17, 29.4%) than in the STING‐high SCLCs (2/14, 14.3%) (Figure 4C), consistent with the observation at transcriptomic level which showed a low degree enrichment of NOTCH signaling in STING‐low SCLC (Figure 4A). Notably, mutations in DNA mismatch repair (MMR) genes were more frequent in STING‐high SCLC, whereas no mutations in these genes were detected in STING‐low SCLC tumors (Figure 4C), further confirming the inactivation of MMR pathway in STING‐high SCLCs (Figure 4B).

### 3.6 Targeting DNA damage response triggers innate immunity in STING‐low SCLC

Recent studies on SCLC showed that the effect of the combination therapy with DDR inhibitors was correlated with the activity of DNA repair response in patients and that DDR inhibitors could trigger STING‐IFN activation in SCLC models.^20^, ^22^ However, the potential effect of the combination of DDR inhibitor and chemotherapy in triggering immune response signaling is yet largely unknown.

**FIGURE 5:**
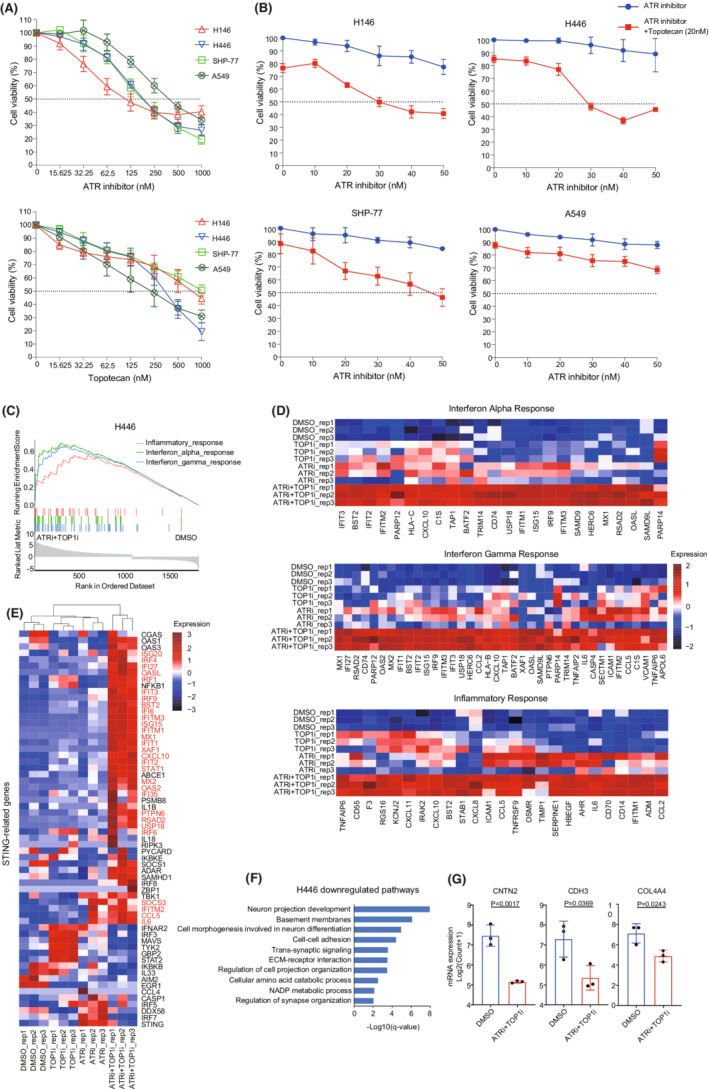
Targeting DNA damage response triggers innate immunity in SCLC cells. (A) Cytotoxicity of ATR inhibitor (M4344) and Topotecan as monotherapy in three SCLC and one NSCLC cell lines. (B) SCLC and NSCLC cells were co‐incubated with the indicated concentrations of Topotecan and ATR inhibitor for 48 h. (C) GSEA of the differentially expressed genes induced by ATR inhibitor and Topotecan in H446 cell line. Shown are three of the top seven most positively regulated “hallmark” signatures. (D) Heatmaps showing expression level of genes involved in three top positively regulated pathways. Color gradation is based on sample‐wise z‐score standardized values. (E) Heatmap showing expression of 62 STING signaling‐related genes in pre‐ and post‐treatment H446 cells. Red font represents significantly upregulated genes in combined treatment group compared with control group. (F) Pathway enriched analysis for downregulated genes in combination therapy group compared with control in H446 cells based on KEGG, Reactome, and GO gene sets. (G) Boxplots of *CNTN2*, *CDH3*, and *COL4A4* expression in H446 cells treated with ATR and TOP1 inhibitors. Statistical significance was determined by unpaired *t*‐test (G).

Here, H146 and H446 cell lines were defined as STING‐low SCLC, whereas SHP‐77 was identified as STING‐intermediate SCLC based on our STING‐related gene signature (Figure S4A). *STING* expression was significantly higher in SHP‐77 than in H146 and H446 cell lines (Figure S4B). Additionally, H146 and SHP‐77 showed high *ASCL1* expression, while H446 was characterized by a high level of *NEUROD1* (Figure S4C). We first tested the cytotoxicity of ATR inhibitor (M4344) and TOP1 inhibitor (Topotecan), as monotherapy in SCLC and NSCLC cell lines. Differential cytotoxicity was observed between cell lines treated with M4344, whereas the toxic effect of Topotecan on SCLC cells is steadily dose‐dependent within a very wide range of concentrations, and highly comparable across all tested SCLC cell lines (Figure 5A). Impressively, we observed that the concurrent treatment with M4344 and Topotecan yielded a robust synergy in repressing cell viability in SCLC cells (Figure 5A,B), in line with previous studies.^20^, ^21^

Next, transcriptomic sequencing was performed to investigate transcriptional changes induced by the combination treatment. Unexpectedly, the combination treatment with M4344 and Topotecan failed to activate immune response in H146 and SHP‐77 cells, while it was capable of activating type I IFN signaling in H446 cells (Figure 5C–E and Figure S4D,E). Indeed, GSEA analysis for H446 cells revealed that the combination of M4344 and Topotecan significantly upregulated interferon‐stimulated genes (ISGs), including genes involved in “Interferon Alpha (IFN‐α) Response”, “Interferon Gamma (IFN‐γ) response”, and “Inflammatory Response” (Figure 5C,D). Moreover, simultaneous inhibition of ATR and TOP1 induced as well a significant increase in the expression of MHC I antigen presentation (*HLA‐B*, *HLA‐C*, and *TAP1*) and most of the STING‐related genes (*CXCL10*, *CCL5*, *ISG15*, and *IFITM1*, etc.) (Figure 5D,E). Additionally, the concurrent treatment with M4344 and Topotecan reduced expression of genes related to neuron projection development (*CNTN2*), cell–cell adhesion (*CDH3*), and ECM‐receptor interaction (*COL4A4*) (Figure 5F,G). These results indicated that the inhibition of ATR and TOP1 could stimulate innate immunity and suppress neuronal differentiation of NE cells.

While in ASCL1‐high expression cell lines (H146 and SHP‐77), treatment with ATR and TOP1 inhibitors induced no immune response signaling activation and pro‐inflammatory cytokines/chemokines expression. Instead, treated H146 and SHP‐77 cells presented respectively up‐regulated expression of genes linked to cell‐growth progression (Figure S4D) and cell cycle and metabolism signaling pathways (Figure S4E). Similarly, in NSCLC cell line (A549), M4344 and Topotecan altered not the expression of ISGs or other immune genes, but upregulated genes involved in p53 transcriptional gene network and NF‐kB signaling (Figure S4F).

Collectively, our findings suggest that concurrent inhibition of ATR and TOP1 in a STING‐low SCLC cell line (H446 cell line) triggered the expression of genes encoding type I IFN signaling and pro‐inflammatory cytokines/chemokines and thus has potential to transform “cold” tumors into “hot” tumors.

## 4 DISCUSSION

SCLC is a lethal and exceptionally aggressive malignancy. Molecular subtypes according to transcriptional profiles have been described in SCLC, and several subtype‐specific therapeutic approaches have been tested for SCLC.^10^, ^11^ However, conflicting observations have been reported regarding SCLC subtypes defined by the expression of transcription factors. For instance, SCLC‐I subtype is described as a tumor with high inflammatory composition, but also as a tumor with immunosuppressive microenvironment, suggesting insufficient understanding of SCLC subtypes and necessity of further cell‐intrinsic and ‐extrinsic characterizations of SCLC subtypes.^11^ Here, by systematic characterization of STING pathway in SCLC, we found that the STING‐high subtype is associated with more abundant immune infiltrates, while the STING‐low subtype exhibits higher NE score and the enrichment of cell cycle pathways. Importantly, we noticed that extrinsic stimuli, such as ATR inhibitor and Topotecan, can trigger the expression of genes encoding type I IFN signaling and pro‐inflammatory cytokines/chemokines only in a subset of SCLC, providing experimental basis of refined treatment strategies for SCLC patients.

*STING* expression is usually suppressed or absent in the majority of cancer types. For instance, *STING* expression was downregulated in gastric cancer and hepatocellular carcinoma compared to corresponding normal tissues, and this lower expression level was correlated with poorer prognosis.^16^, ^43^ We observed intrinsic low *STING* expression, both at mRNA and protein levels, in SCLC compared to normal lung tissues, LUSC, and LUAD, suggesting a strong suppression of *STING* expression in SCLC. Additionally, it has been shown that the expression of other members of STING pathway is also suppressed in SCLC, such as *IFI16* and *DDX60*. These findings suggest that these STING‐related molecules might act as tumor suppressors in SCLC, but further functional analyses are required to assess whether these factors contribute to SCLC tumorigenesis. Interestingly, despite the STING deficiency in SCLC, the expression of cGAS sustained, indicating the importance of cGAS‐independent pathways in SCLC. For instance, *IFI16* has been reported to activate STING in a cGAS‐independent manner.^44^

While SCLC tumors exhibit general low level of *STING* expression, we identified three distinct SCLC subtypes based on the expression of a STING‐signature (STING‐high, STING‐intermediate, and STING‐low).

When we tried to correlate genomic features of SCLC tumors to the STING activation levels, we found that STING‐low tumors had higher mutation frequency in NOTCH‐pathway genes compared with STING‐high tumors. *NOTCH* mutations may cause important switch of the function of STING pathway regulators.

The activity of STING pathway is subjected to epigenetic modifications and subsequently acquires altered functions. For example, epigenetic silencing of STING‐related genes is not only a notable mechanism of STING signaling dysfunction in melanoma but also plays a role in impaired tumor antigen presentation and recognition by tumor‐infiltrating lymphocytes.^45^ Epigenetic reprogramming by DNMT1 and/or EZH2 inhibitors can restore STING expression in NSCLC cells.^46^ Yet, further work is still needed to understand the epigenetic regulation of STING‐pathway activity in SCLC.

Activated STING pathway promotes production of type I IFNs, in turn, boosts the antitumor immunity by enhancing antigen presentation, and recruits T cell into tumor site.^17^ In line with this, we observed that STING‐high SCLC exhibited an inflammation phenotype with abundant immune cell infiltration and high expression of MHC and immune checkpoints genes. Notably, STING‐high SCLC does not exactly coincide with the SCLC‐I subtype proposed by Gay et al.,^11^ suggesting that the STING‐subtypes are not a replicate of SCLC subtypes defined by transcription factors.

An important observation is STING‐low SCLC exhibited activation of pathways associated with DNA replication and cell cycle, and their activation was associated with high replication stress.^20^, ^41^ SCLCs with high replication stress were more likely to respond to DDR inhibitors.^20^ DDR inhibitors, such as PARP1 and CHK1 inhibitors, can induce cytosolic DNA which initiate cGAS‐STING‐mediated interferon response in SCLC.^22^ We, therefore, hypothesized that targeting DDR by inhibiting ATR together with chemotherapy might have the potential to activate cGAS‐STING‐IFN signaling in SCLC. In our study, ATR and TOP1 inhibitors, trigger the expression of genes encoding type I IFN signaling and pro‐inflammatory cytokines/chemokines in a STING‐low SCLC cell line (H446). Unexpectedly, we did not observe significant changes in cytokine and chemokine expression in H146 and SHP‐77 cell lines upon the combination treatment of ATR and TOP1 inhibitors. The differential activation of type I IFN signaling in two STING‐low SCLC cell lines might due to the cellular and molecular heterogeneities between two cell lines. H146 cell line was derived from bone marrow (bone metastasis of SCLC cells) of a SCLC patient, whereas H446 cell line was derived from the pleural fluid of a SCLC patient. Moreover, H146 cells express high levels of *ASCL1* but H446 cells express high level of *NEUROD1*. These two transcription factors regulate distinct biological processes in SCLC.^47^ However, whether the failure in activating immune response in STING‐low SCLC (H146) is related to high *ASCL1* expression requires further verification. Another one possible explanation is, we have only evaluated the activation of type I IFN response signaling in the treated SCLC cells in vitro with absence of immune cells. While, in the setting with a complete immune system in vivo, DDR inhibitor‐derived DNA damage may induce type I IFN response in dendritic cells other than cancer cells.^45^ Nevertheless, the durable clinical benefit from the combination treatment of ATR and TOP1 inhibitors in STING‐low SCLC and the corresponding molecular mechanisms need to be further verified and explored.

Despite recent advances in the immunotherapy of patients with SCLC, the resulting survival rates remain very poor. Our data provide strong evidence that combining ATR and TOP1 inhibitors in cancer cells triggers robust immune response signaling and secretion of pro‐inflammatory factors, and thus might transform “cold” tumors into “hot” tumors, which are more likely to respond to systemic immunotherapy.

Our study has few limitations. First, given the complex interactions between tumor and immune cells, computational tools for the characterization of the immunophenotypes of the tumors based on the gene signatures can only suggest the relative composition of immune cells but cannot quantify absolute infiltration of each type of immune cell. Further studies need to quantitatively depict the tumor immune microenvironment remodeled by targeting DDR. Second, further in vivo experiments need to be performed to verify the effects of DDR inhibitors on increasing immunogenicity of SCLC.

In conclusion, we identify distinct STING‐related SCLC subtypes and each subtype demonstrates distinct patterns of immune infiltrates and activated biological processes. We aim to provide a novel molecular classification of SCLC which may benefit patient stratification as well as facilitate design refined therapeutic approaches. Molecular assessment of pre‐ and post‐treatment SCLC cell lines in this study provides a framework to understand the molecular mechanism of duo‐inhibition of ATR and TOP1 and paves a way for the development of new therapeutic strategy for SCLC.

## AUTHOR CONTRIBUTIONS

X.T.L. and J.H. conceived the original idea; X.T.L. performed all analysis; N.B.L. and G.R.L. performed IHC staining and analysis; Y.J.L., S.Q.W., and Z.W.Z. provided critical feedback and discussion; L.Y.D. collected clinical data; X.T.L. and J.H. wrote the manuscript with input from all authors. All authors have read and agreed to the published version of the manuscript.

## FUNDING INFORMATION

This work was supported by Guangzhou Science Technology and Innovation Commission [grant number 201807010107], the Natural Science Foundation of Guangdong [grant number 2021A1515012123], and the Fundamental Research Funds for the Central Universities, SCUT [grant number X2YXD2172070].

## CONFLICT OF INTEREST

The authors declare no potential conflicts of interest.

## ETHICAL APPROVAL STATEMENT

This study was reviewed and approved by the Ethical Review Committee for Biomedical Research, Guangzhou First People's Hospital. The study design and all procedures were conducted in accordance with the Declaration of Helsinki.

## Supporting information


Figures S1–S4
Table S1–S2

## Data Availability

Data availability statement The raw RNA‐seq data generated in this study are publicly available in Genome Sequence Archive for Human at HRA001720.
