# Supplementary material for: Immunogenicity of small‐cell lung cancer associates with STING pathway activation and is enhanced by ATR and TOP1 inhibition
Source: Cancer Med. 2022 Aug 11;12(4):4864–81. doi: 10.1002/cam4.5109 (PMC9972012; doi:10.1002/cam4.5109)
Supplement: Supplementary file 1 — Figures S1–S4 Table S1–S2 [file CAM4-12-4864-s001.docx]

**Supplementary Figures and Tables**

**Supplementary Figures**

**Fig. S1**


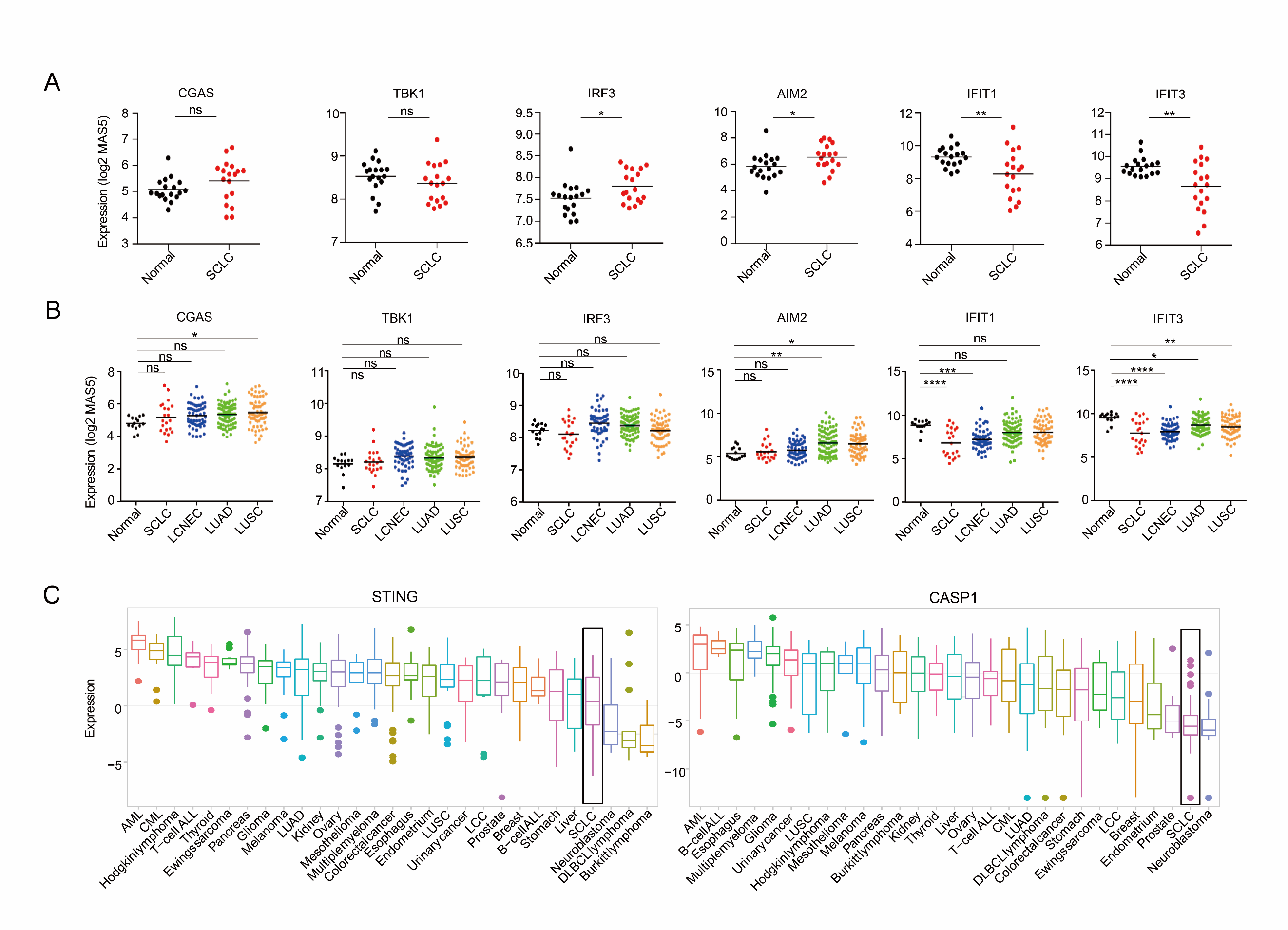


**Fig. S1| Expression of STING signaling-related genes in SCLC. A.** Relative mRNA expression of STING signaling-related genes in SCLC and paired normal lung tissues. **B.** Relative mRNA expression of STING signaling-related genes among different histological subtypes in lung. **C.** Relative mRNA expression of STING and CASP1 in cancer cell lines from CCLE dataset. P values were calculated by unpaired t test (**A**) and One-way ANOVA (**B**). P values of statistical significance are represented as *P < 0.05, **P < 0.01, ***P < 0.001, and ****P < 0.0001, ns, not significant.

Fig. S2


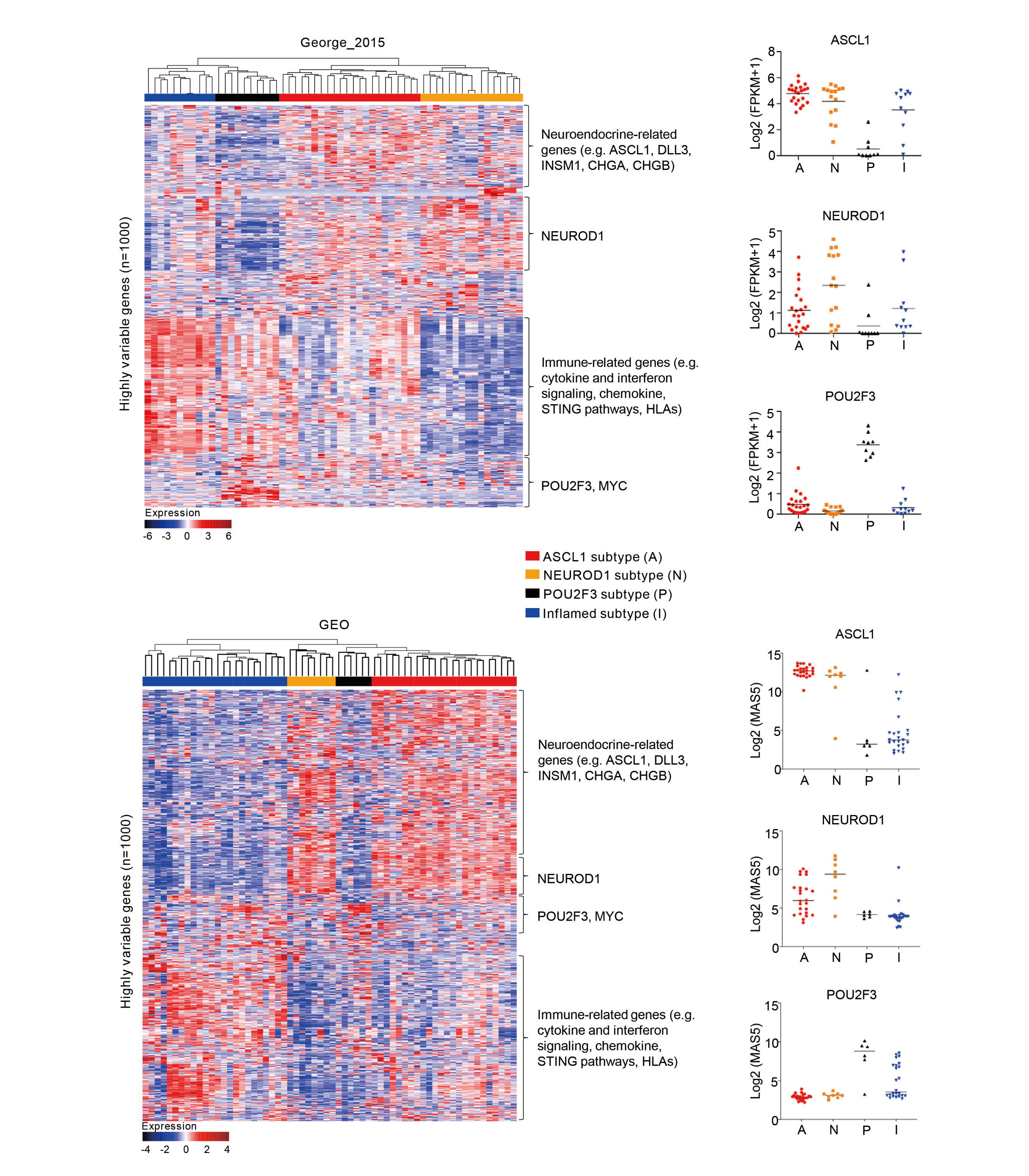


**Fig. S2|** **Transcription factor-defined SCLC subtypes in our cohorts.** Highly variable genes were used to identify SCLC clusters according to reported method by Gay et al (Cancer Cell. 2021;39:346-360.e7), consistent with molecular feature of transcription factor-defined (ASCL1, NEUROD1, and POU2F3) subtypes.

Fig. S3


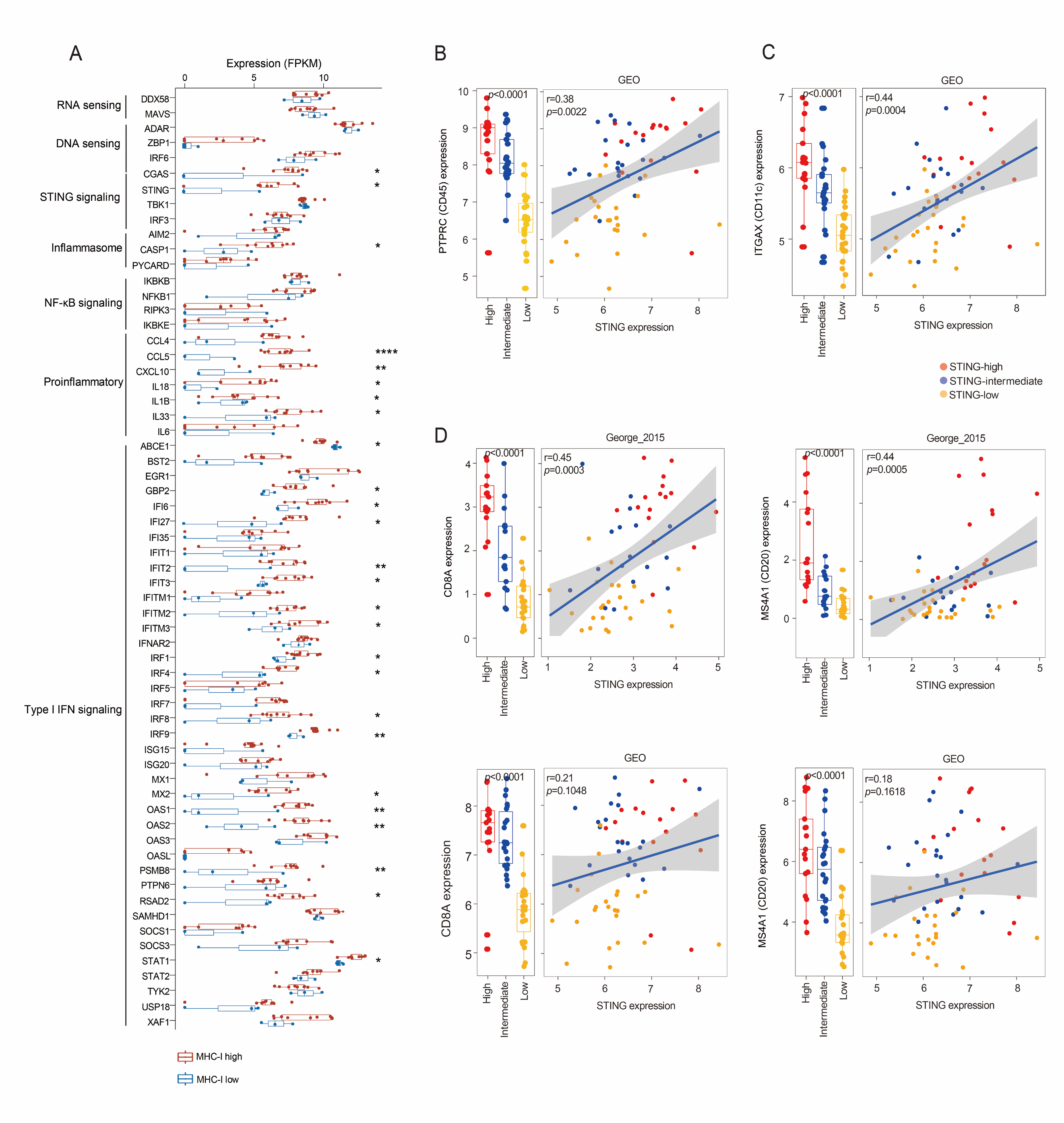


**Fig. S3| The relationship between STING-related genes expression and immune-related genes expression in SCLC. A**. The comparation of STING-related genes expression between MHC-high and MHC-low SCLC patients (from GSE168266 dataset). **B-D**. Box whisker plots show the levels of four marker genes of immune cell (PTPRC, ITGAX, CD8A, and MS4A1) among different STING subtypes. Scatterplots show the relationship between expression of STING and expression of four marker genes. Color for scatterplot symbols reflects different STING subtypes. P values were calculated by unpaired t test (**A**) or One-way ANOVA test (**B**, **C**, and **D** for box whisker plots) or Pearson correlation analysis (**B**, **C**, and **D** for scatterplots). P values of statistical significance are represented as *P < 0.05, **P < 0.01, ***P < 0.001, and ****P < 0.0001.

Fig. S4


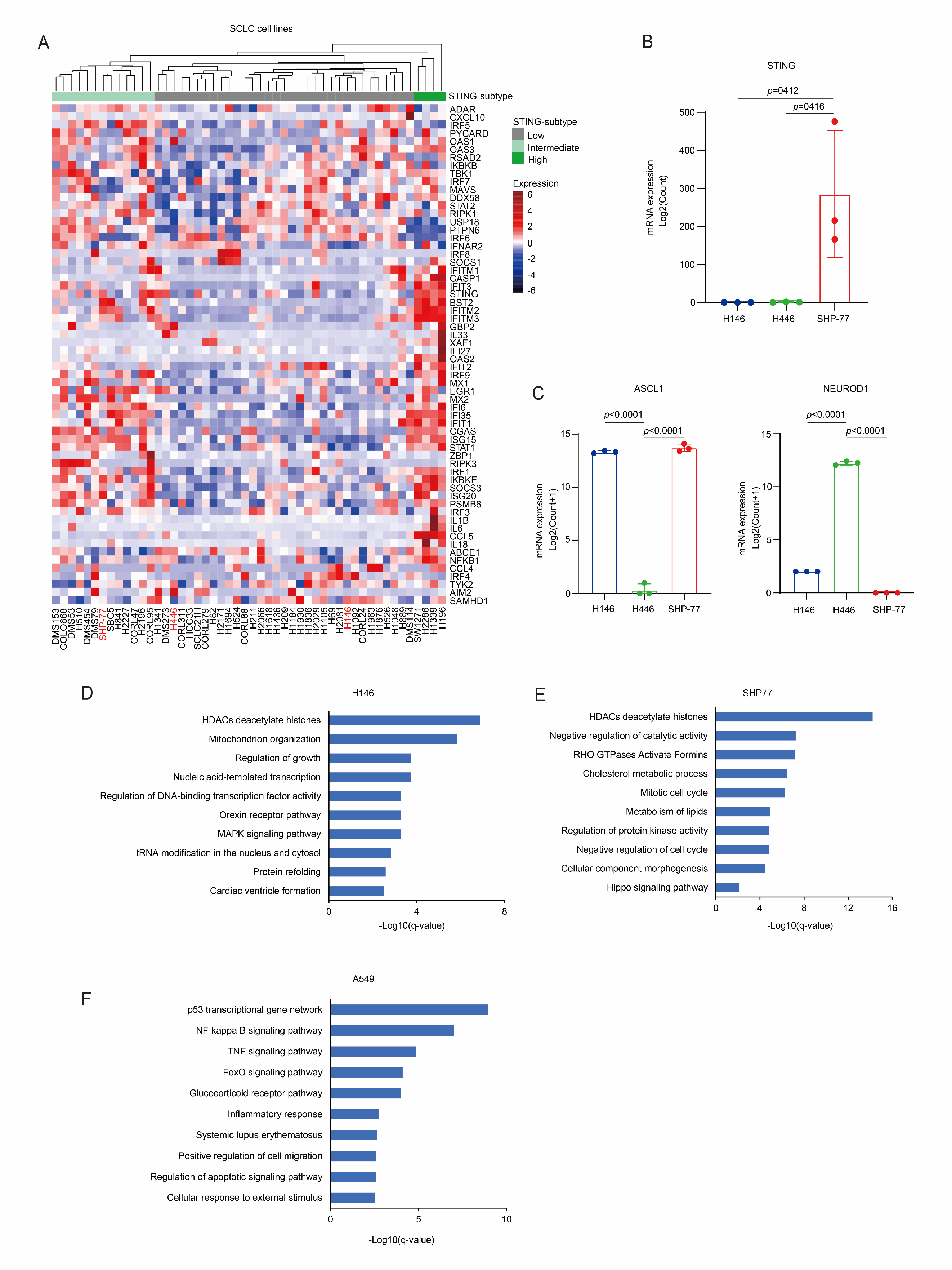


**Fig. S4|** **A**. Hierarchical clustering analysis was performed to cluster SCLC samples based on STING signaling-related genes in 50 SCLC cell lines from CCLE dataset. **B**. Expression level of STING gene among three SCLC cell lines. **C**. Relative mRNA expression of ASCL1 and NEUROD1 among three SCLC cell lines. **D-F**. Pathway enriched analysis for upregulated genes in combination therapy group compared with control group in H146 (**D**), SHP-77 (**E**), and A549 (**F**) cell lines based on KEGG, Reactome, and GO gene sets. P values were calculated by unpaired t test (**B** and **C**).

**Supplementary Tables**

**Table S1| The gene list of STING signaling-related genes.**

| Symbol | Signaling |
| --- | --- |
| DDX58 | dsRNA sensing |
| MAVS | dsRNA sensing |
| ADAR | dsDNA sensing |
| ZBP1 | dsDNA sensing |
| IRF6 | dsDNA sensing |
| CGAS | dsDNA sensing |
| STING | STING signaling |
| TBK1 | STING signaling |
| IRF3 | STING signaling |
| AIM2 | Inflammasome |
| CASP1 | Inflammasome |
| PYCARD | Inflammasome |
| IKBKB | NF-KB signaling |
| IKBKE | NF-KB signaling |
| NFKB1 | NF-KB signaling |
| RIPK3 | NF-KB signaling |
| CCL4 | proinflammatory |
| CCL5 | proinflammatory |
| CXCL10 | proinflammatory |
| IL18 | proinflammatory |
| IL1B | proinflammatory |
| IL33 | proinflammatory |
| IL6 | proinflammatory |
| ABCE1 | Type I IFN signaling |
| BST2 | Type I IFN signaling |
| EGR1 | Type I IFN signaling |
| GBP2 | Type I IFN signaling |
| IFI27 | Type I IFN signaling |
| IFI35 | Type I IFN signaling |
| IFI6 | Type I IFN signaling |
| IFIT1 | Type I IFN signaling |
| IFIT2 | Type I IFN signaling |
| IFIT3 | Type I IFN signaling |
| IFITM1 | Type I IFN signaling |
| IFITM2 | Type I IFN signaling |
| IFITM3 | Type I IFN signaling |
| IFNAR2 | Type I IFN signaling |
| IRF1 | Type I IFN signaling |
| IRF4 | Type I IFN signaling |
| IRF5 | Type I IFN signaling |
| IRF7 | Type I IFN signaling |
| IRF8 | Type I IFN signaling |
| IRF9 | Type I IFN signaling |
| ISG15 | Type I IFN signaling |
| ISG20 | Type I IFN signaling |
| MX1 | Type I IFN signaling |
| MX2 | Type I IFN signaling |
| OAS1 | Type I IFN signaling |
| OAS2 | Type I IFN signaling |
| OAS3 | Type I IFN signaling |
| OASL | Type I IFN signaling |
| PSMB8 | Type I IFN signaling |
| PTPN6 | Type I IFN signaling |
| RSAD2 | Type I IFN signaling |
| SAMHD1 | Type I IFN signaling |
| SOCS1 | Type I IFN signaling |
| SOCS3 | Type I IFN signaling |
| STAT1 | Type I IFN signaling |
| STAT2 | Type I IFN signaling |
| TYK2 | Type I IFN signaling |
| USP18 | Type I IFN signaling |
| XAF1 | Type I IFN signaling |

**Table S2| The gene lists of NE/non-NE genes, EMT genes, and Ferroptosis genes.**

| NE | EMT | Ferroptosis |
| --- | --- | --- |
| ADGRG6 | CADM1 | ACSL4 |
| AHNAK | COL1A2 | AKR1C1 |
| ANXA1 | ACTA2 | AKR1C2 |
| ANXA3 | DKK1 | AKR1C3 |
| AP3B2 | COL4A2 | ALOX15 |
| BEX1 | CD44 | ALOX5 |
| CAV1 | CXCL1 | ALOX12 |
| CAV2 | BMP1 | ATP5MC3 |
| CHGA | BGN | CARS |
| CHGB | DPYSL3 | CBS |
| CHRNB2 | CDH11 | CD44 |
| CRMP1 | COMP | CHAC1 |
| CYR61 | CDH6 | CISD1 |
| DPYSL5 | COL6A2 | CS |
| EMP1 | CTGF | DPP4 |
| EPHA2 | COL3A1 | FANCD2 |
| GNAO1 | ECM1 | GCLC |
| GNG4 | COPA | GCLM |
| GPRC5A | CALD1 | GLS2 |
| GPX8 | CALU | GPX4 |
| HFE | CD59 | GSS |
| IFITM3 | COL5A3 | HMGCR |
| IL18 | COL16A1 | HSPB1 |
| INSM1 | CRLF1 | CRYAB |
| ITGB4 | COL5A1 | LPCAT3 |
| KIF1A | CXCL12 | MT1G |
| KIF5C | BASP1 | NCOA4 |
| LGALS3 | COL6A3 | PTGS2 |
| MAPK8IP1 | CYR61 | RPL8 |
| MYOF | CXCL6 | SAT1 |
| NABP1 | ADAM12 | SLC7A11 |
| NCAM1 | DAB2 | FDFT1 |
| NT5E | COL12A1 | TFRC |
| OSMR | AREG | TP53 |
| PGBD5 | CAP2 | EMC2 |
| RAB39A | COL8A2 | AIFM2 |
| RHBDF1 | COL4A1 | PHKG2 |
| RUNDC3A | BDNF | HSBP1 |
| S100A16 | COL7A1 | ACO1 |
| SCAMP5 | DCN | FTH1 |
| SCG3 | APLP1 | STEAP3 |
| SNAP25 | COL5A2 | NFS1 |
| SOGA3 | COL11A1 | ACSL3 |
| SYP | CTHRC1 | ACACA |
| TGFBR2 | COL1A1 | PEBP1 |
| TMEM198 | DST |  |
| TUBB2B | CAPG |  |
| UNC13A | ANPEP |  |
| WWTR1 | CDH2 |  |
| YAP1 | ABI3BP |  |
|  | ECM2 |  |
|  | EDIL3 |  |
|  | EFEMP2 |  |
|  | ELN |  |
|  | EMP3 |  |
|  | ENO2 |  |
|  | FAP |  |
|  | FAS |  |
|  | FBLN1 |  |
|  | FBLN2 |  |
|  | FBLN5 |  |
|  | FBN1 |  |
|  | FBN2 |  |
|  | FERMT2 |  |
|  | FGF2 |  |
|  | FLNA |  |
|  | FMOD |  |
|  | FN1 |  |
|  | FOXC2 |  |
|  | FSTL1 |  |
|  | FSTL3 |  |
|  | FUCA1 |  |
|  | FZD8 |  |
|  | GADD45A |  |
|  | GADD45B |  |
|  | GAS1 |  |
|  | GEM |  |
|  | GJA1 |  |
|  | GLIPR1 |  |
|  | GLT25D1 |  |
|  | GPC1 |  |
|  | GPX7 |  |
|  | GREM1 |  |
|  | HTRA1 |  |
|  | ID2 |  |
|  | IGFBP2 |  |
|  | IGFBP3 |  |
|  | IGFBP4 |  |
|  | IL15 |  |
|  | IL32 |  |
|  | IL6 |  |
|  | IL8 |  |
|  | INHBA |  |
|  | ITGA2 |  |
|  | ITGA5 |  |
|  | ITGAV |  |
|  | ITGB1 |  |
|  | ITGB3 |  |
|  | ITGB5 |  |
|  | JUN |  |
|  | LAMA1 |  |
|  | LAMA2 |  |
|  | LAMA3 |  |
|  | LAMC1 |  |
|  | LAMC2 |  |
|  | LEPRE1 |  |
|  | LGALS1 |  |
|  | LOX |  |
|  | LOXL1 |  |
|  | LOXL2 |  |
|  | LRP1 |  |
|  | LRRC15 |  |
|  | LUM |  |
|  | MAGEE1 |  |
|  | MATN2 |  |
|  | MATN3 |  |
|  | MCM7 |  |
|  | MEST |  |
|  | MFAP5 |  |
|  | MGP |  |
|  | MMP1 |  |
|  | MMP14 |  |
|  | MMP2 |  |
|  | MMP3 |  |
|  | MSX1 |  |
|  | MXRA5 |  |
|  | MYL9 |  |
|  | MYLK |  |
|  | NID2 |  |
|  | NNMT |  |
|  | NOTCH2 |  |
|  | NT5E |  |
|  | NTM |  |
|  | OXTR |  |
|  | PCOLCE |  |
|  | PCOLCE2 |  |
|  | PDGFRB |  |
|  | PDLIM4 |  |
|  | PFN2 |  |
|  | PLAUR |  |
|  | PLOD1 |  |
|  | PLOD2 |  |
|  | PLOD3 |  |
|  | PMEPA1 |  |
|  | PMP22 |  |
|  | POSTN |  |
|  | PPIB |  |
|  | PRRX1 |  |
|  | PRSS2 |  |
|  | PTHLH |  |
|  | PTX3 |  |
|  | PVR |  |
|  | QSOX1 |  |
|  | RGS4 |  |
|  | RHOB |  |
|  | SAT1 |  |
|  | SCG2 |  |
|  | SDC1 |  |
|  | SDC4 |  |
|  | SERPINE1 |  |
|  | SERPINE2 |  |
|  | SERPINH1 |  |
|  | SFRP1 |  |
|  | SFRP4 |  |
|  | SGCB |  |
|  | SGCD |  |
|  | SGCG |  |
|  | SLC6A8 |  |
|  | SLIT2 |  |
|  | SLIT3 |  |
|  | SNAI2 |  |
|  | SNTB1 |  |
|  | SPARC |  |
|  | SPOCK1 |  |
|  | SPP1 |  |
|  | TAGLN |  |
|  | TFPI2 |  |
|  | TGFB1 |  |
|  | TGFBI |  |
|  | TGFBR3 |  |
|  | TGM2 |  |
|  | THBS1 |  |
|  | THBS2 |  |
|  | THY1 |  |
|  | TIMP1 |  |
|  | TIMP3 |  |
|  | TNC |  |
|  | TNFAIP3 |  |
|  | TNFRSF11B | |
|  | TNFRSF12A | |
|  | TPM1 |  |
|  | TPM2 |  |
|  | TPM4 |  |
|  | VCAM1 |  |
|  | VCAN |  |
|  | VEGFA |  |
|  | VEGFC |  |
|  | VIM |  |
|  | WIPF1 |  |
|  | WNT5A |  |
